# Inclusive innovation in telehealth

**DOI:** 10.1038/s41746-020-0296-5

**Published:** 2020-06-25

**Authors:** Kimberly Noel, Brooke Ellison

**Affiliations:** 1 Stony Brook Renaissance School of Medicine, Stony Brook University Hospital, 101 Nicolls Rd, Stony Brook, NY United States; 2 Stony Brook School of Health Technology and Management, Stony Brook University Hospital, 101 Nicolls Rd, Stony Brook, NY United States

**Keywords:** Medical ethics, Research management

## Abstract

It has been 30 years since the passage of the Americans with Disabilities Act and technological development has drastically changed the future for those with disabilities. As healthcare evolves toward promoting telehealth and patient-centered care, leaders must embrace persons with disabilities and caregivers as valued partners in design and implementation, not as passive “end-users”. We call for a new era of inclusive innovation, a term proposed in this publication to describe accessible technological design for all. The next 30 years of the ADA leading to year 2050, should reflect a new era of access, whereby digital health surmounts geographic, social, and economic barriers toward an inclusive virtual society.

This year, 2020 marks the 30th anniversary of the Americans with Disabilities Act (ADA), a piece of civil rights legislation that positively changed the lives of people with disabilities across the country. Despite updates made to the legislation since its original passage, the ADA initially focused on pressing issues of accessibility in physical structures and architecture. While this need remains true today, technology has become a more prominent issue. This is particularly important during the Coronavirus pandemic, where rapid innovations in telehealth have a large marginal impact for people with disabilities. Participation from this population remains woefully limited in technological development, despite the impact it can have on their quality of life. The next 30 years of the ADA should reflect a new era, one of “inclusive innovation”, a term introduced in this text, to describe technological development in telehealth, where those with disabilities and caregivers are seen as strategic partners. By acknowledging the invaluable insight from persons traditionally overlooked, inclusive innovation will establish better design and user experience for all.

After years of slow adoption, telehealth, defined here as the use of electronic information and communication technologies in healthcare, has seen overwhelming growth during the Coronavirus pandemic. Along with increased utilization, telehealth has expanded beyond live video technologies, including remote patient monitoring, mobile health, asynchronous and emerging technologies. These diverse modalities hold promise in enhancing healthcare beyond episodic in-person care. People with disabilities often have complex medical and healthcare access needs for which telehealth is particularly well-suited, especially in management of chronic diseases. According to the WHO’s *World Report*, chronic conditions are not only highly prevalent but are often manifested in complex interrelationships, which poses unique challenges for those with disabilities^1^. Telehealth, especially with remote patient monitoring, can enhance surveillance of chronic conditions, improve quality of care and access to care. Telehealth reduces the need for financially and physically burdensome travel that is challenging for this population and caregivers. Telehealth promises reduced adverse health outcomes, care coordination needs, and may even reduce risk of long-term hospitalization or institutionalization^2^. Furthermore, telehealth may provide protection from iatrogenic infections, particularly during high prevalence periods of influenza or outbreaks such as Coronavirus^3^.

While telehealth will be the future of healthcare for everyone, advances in the field must prevent exacerbating existing social and health disparities. According to the Pew Research Center, Americans with disabilities experience critical access barriers due to technologies unable to accommodate their physical needs, and disparate socioeconomic challenges that result in a dramatic decrease in likelihood of going online. When the internet provides the most immediate medium to establish communication between physicians and patients, this technological disparity can be the basis of a concomitant health disparity. What is more, people with disabilities are 20% less likely than people without disabilities to own a computer, smartphone or tablet—the mechanisms by which much telehealth innovation is delivered and telehealth information is recorded^4^.

Despite the lack of access to technology that many people with disabilities face, there is a strong desire among this population to be a part of this healthcare technological revolution. Data published by CMS showed that between 2014 and 2016, there was a 37.7% increase in the number of beneficiaries with disabilities using telehealth, and a 53.7% increase in the total services these beneficiaries used^5^. In 2016, persons with disabilities accounted for 65% (58,406) of beneficiaries using telehealth, using over 66% (182,858) of all telehealth services^5^. Within these data, some interesting insights can be gleaned. The data represents high utilization for those under age 65 who are eligible for Medicare due to disability specifically. The report also describes a sizable “dual eligible” population insured by both Medicare and Medicaid, implying that they may experience financial insecurity but have been able to utilize telehealth services, nonetheless. From these data, an important claim can be made: people with disabilities, especially younger adults who will continue to experience years of healthcare needs, see the value in telehealth services and will seek these services even when resources are low. Enhancing telehealth access to this population is essential.

There is a strong moral imperative to inclusive innovation—we, as a society, are better off when everyone can take part in it and our societal developments must take place in a way that reflects that belief. People with disabilities have been historically removed from the site of conversations. This has led to a technological frontier that has been largely absent of the voices, insights, and experiences of people with disabilities. However, in addition to the humanitarian necessity behind inclusive innovation, serving the needs of the disabled also makes sound financial sense^6^. According to market research, the disability market is sizable, representing 1.3 billion people with a projected $1.2 trillion in annual disposable income^7^. Ineffective user experience (UX) research is self-reportedly a trillion-dollar issue per the tech industry^8^. Especially given our aging population who will experience disability at a greater prevalence, innovations for people with disabilities have a large economic and political potential that promises significant returns on investment. Technologies that increase user independence, such as those related to the “internet of things” can be invaluable for people with disabilities, especially those with mobility impairments, but they are also highly coveted by the non-disabled population, creating a universal market driven by the needs of those who are most vulnerable^9,10^. In a framework that mirrors the societal benefits of universal design, what is useful for people with disabilities is also often useful for most others.

Achieving inclusive innovation in telehealth, means changing the practice of medicine. The advancements of telehealth, especially for people with disabilities, requires influencing evidence-based guidelines, facilitating large clinical trials, and testing new modes of healthcare delivery^11^. There is a saying among accessibility advocates “nothing about us without us”, which conveys the importance of representing persons with disabilities in services tailored for the population^12^. Inclusive innovation furthers this argument advocating for a “nothing without us” approach to emphasize the importance of inclusion of those with disabilities in technological development for all end users. This view advocates for comprehensive engagement in technological development, clinical trial design, and participation, as well as consumer experience reporting and testing. Large *digiceutical* trials should evaluate meaningful health metrics for usability and acceptability of telehealth technologies including those with disabilities. Just as telehealth must have an eye toward inclusion for people with disabilities, so, too, must innovation in general. Unsurprisingly, what is beneficial for people with disabilities also turns out to be beneficial for everyone.

Major stakeholders in the technology sector have only just begun to realize and learn from the experiences of people with disabilities, and their innovation has accelerated as a result. For instance, even in their most recent stages, smart-home assistant devices like Amazon’s Alexa and the Google Assistant have generated enormous amounts of independence and opportunity to people with disabilities^13,14^. As the market has grown and has included the general population, more capacity has been generated for enhanced, advanced innovation geared even more specifically for the lives of people with disabilities. Google’s Projects: Diva, Euphonia, and Hello Morse all have pursued the development of increased interfaces through which people with disabilities can engage with technology, as has Microsoft’s pledge to dedicate 25 million for artificial intelligence (AI) in accessibility^15–18^.

Powerful collaborations are essential in creating equity in technological innovation and adoption. Now is the time for technology innovators of all kinds to seize upon this intersection of need, demand, and opportunity. There is tremendous potential for a mutually beneficial collaboration between people with disabilities, technology giants, and smaller innovators alike. The current climate is optimal for social entrepreneurship, in which industry and disability advocates unite to research and develop in a way that involves everyone: true inclusive innovation.
